# ICG-only Sentinel Lymph Node Biopsy in Breast Cancer: A Prospective UK Cohort Demonstrating Consistent Detection Across Patient and Tumour Characteristics—A Follow-on to the INFLUENCE Trial

**DOI:** 10.1245/s10434-026-20316-w

**Published:** 2026-07-29

**Authors:** Vassilis Pitsinis, Nadia Khitab, Lee Jordan, Alessio Vinci, Rahul Kanitkar, Emad Elseedawy, Jennifer Smith

**Affiliations:** University of Dundee and NHS Tayside, Dundee, Scotland

**Keywords:** Indocyanine green, Sentinel lymph node biopsy, Breast cancer, Near-infrared fluorescence, Lymphatic mapping, Single-tracer technique, Axillary staging, Fluorescence-guided surgery

## Abstract

**Background:**

Sentinel lymph node biopsy (SLNB) using dual tracer techniques remains the gold standard for axillary staging in early breast cancer. However, reliance on radioisotope mapping introduces logistical complexity, cost, and radiation exposure. Indocyanine green (ICG) fluorescence imaging has emerged as a potential single-tracer alternative, but concerns remain regarding its reliability across heterogeneous patient populations.

**Methods:**

A prospective single-centre cohort study was conducted including 300 consecutive SLNB procedures across 292 unique patients using ICG as the sole tracer. The primary endpoints were nodal procedural detection rate and ICG nodal detection rate. Secondary outcomes included: nodal positivity, number of nodes retrieved, operative time, the influence of patient, tumour, and procedural variables. Statistical analysis included linear and logistic regression and correlation analysis.

**Results:**

Procedural nodal detection was achieved in 99.7% (299/300) of procedures, while ICG fluorescent nodal detection was achieved in 97.7% (293/300). Median surgeon–pathologist nodal concordance was 100% (IQR 50–100%), with complete count agreement in 50.7% of procedures. Detection was not influenced by age, body mass index (BMI), tumour size, or procedure type. Operative time increased significantly with BMI (*p* = 0.0118) and breast-conserving surgery (*p* < 0.0001).

**Conclusions:**

This large UK prospective series demonstrates that ICG-only fluorescence guidance is safe, efficient, and reproducible, with high procedural and nodal detection rates. Naked-eye visibility of ICG was observed in a subset of cases, further supporting its practicality. Performance was consistent across surgical procedures and patient BMI. These findings support broader adoption of ICG as a single tracer for SLNB.

Sentinel lymph node biopsy (SLNB) has become the standard approach for axillary staging in early breast cancer, allowing accurate nodal assessment while minimising the morbidity associated with axillary lymph node dissection.^1–3^ Traditionally, SLNB is performed using a dual tracer technique combining technetium-99m-labelled radioisotope and blue dye. While highly effective, this approach requires coordination with nuclear medicine services, introduces radiation exposure, and adds logistical and economic burden to surgical pathways.^4,5^

Indocyanine green (ICG) fluorescence imaging has emerged as an alternative technique that enables real-time visualisation of lymphatic drainage without reliance on radioactive tracers. Using near-infrared imaging systems, ICG allows surgeons to directly visualise lymphatic channels and sentinel nodes intraoperatively. Early feasibility studies demonstrated promising detection rates and subsequent systematic reviews and meta-analyses have suggested that ICG may be comparable to traditional dual tracer methods.^6–13^

Despite these encouraging findings, several concerns remain. There is limited prospective evidence evaluating the performance of ICG as a sole tracer across diverse patient populations. Factors, such as increased body mass index (BMI), tumour size, and surgical approach may influence lymphatic mapping or technical accessibility, potentially affecting detection reliability. Furthermore, most existing studies are small, retrospective, or involve hybrid tracer approaches.

The present study addresses these limitations through a prospective evaluation of 300 consecutive SLNB procedures using ICG alone. The primary goal was to assess the nodal procedural detection rate and the ICG nodal detection rate. Secondary objectives included evaluating surgeon-pathologist nodal concordance, operative efficiency, and the influence of patient and tumour characteristics on performance. We hypothesised that ICG would demonstrate consistent detection independent of patient variability, while operative efficiency would be influenced by anatomical and procedural factors.

## Methods

This study was designed as a prospective, single-centre observational cohort study conducted within a tertiary breast surgery unit in the United Kingdom, including all consecutive patients undergoing sentinel lymph node biopsy for invasive breast cancer during the study period. The study was conducted under institutional governance in accordance with the principles of the World Medical Association Declaration of Helsinki, with prospective data collection and analysis performed using a predefined statistical analysis plan developed in collaboration with the Tayside Clinical Trials Unit.

A total of 292 patients undergoing sentinel lymph node biopsy using indocyanine green as the sole tracer were included. Eight of these patients had bilateral procedures, resulting in a total of 300 consecutive procedures. Eligible patients had histologically confirmed invasive breast cancer, a clinically node-negative axilla, and were scheduled for upfront surgical management with sentinel lymph node biopsy. Exclusion criteria included known hypersensitivity to indocyanine green, pregnancy, significant hepatic dysfunction, inflammatory breast cancer, and receipt of neoadjuvant chemotherapy in the presence of known nodal disease. No patients were excluded because of missing data, and complete datasets were available for all analyses.

All procedures were performed using a standardised protocol. Indocyanine green (Verdye^®^, Diagnostic Green GmbH, Germany) was prepared according to the manufacturer’s instructions. A total dose of 2 mg of ICG was administered following dilution with sterile water for injection. One millilitre was injected intradermally and one millilitre subcutaneously in the peri-areolar region, followed by gentle massage to facilitate lymphatic uptake. Sentinel lymph node identification was performed using the SPY-PHI near-infrared fluorescence imaging system (Stryker Corporation, Kalamazoo, MI), allowing real-time visualisation of lymphatic drainage pathways and nodal uptake. All fluorescent nodes were excised and sent for pathological analysis, while additional palpable nodes were removed at the surgeon’s discretion.

The first primary endpoint was the nodal procedural detection rate, defined as successful identification and removal of at least one sentinel lymph node. Failure was defined as the absence of sentinel node identification requiring alternative axillary sampling. The second primary endpoint was the ICG fluorescent nodal detection rate, defined as the proportion of cases where fluorescent nodes were identified during surgery.

Secondary endpoints included surgeon–pathologist nodal concordance, which was defined as the ratio of the smaller of the surgeon-reported and pathology-confirmed node counts to the larger count, expressed as a percentage. A concordance of 100% indicated complete agreement between intraoperative and pathological node counts, whereas lower values reflected increasing discrepancy between the two assessments; nodal positivity, defined as the presence of metastatic disease on histopathology; operative efficiency, measured as the time from incision to first sentinel node identification; visible fluorescence, defined as detection of nodes without imaging assistance; and the presence of residual fluorescent nodes following completion of the procedure.

Derived variables included categorisation of nodal detection rates from 0 to 100%, agreement between surgeon-reported and pathology-confirmed node counts and procedure type, categorised as breast-conserving surgery or mastectomy. Statistical analysis was performed using SAS. Continuous variables were summarised by using mean, standard deviation, median, and interquartile range, while categorical variables were presented as counts and percentages. Linear regression models were used to assess factors influencing nodal detection rate and operative time, whereas logistic regression models were applied to binary outcomes such as procedure type. Both univariate and multivariable models were constructed, including age, body mass index, tumour size, procedure type, and operative time as covariates. Correlation analyses were performed by using both Spearman’s and Pearson’s methods to assess relationships between variables. Statistical significance was defined as a *p* value < 0.05, and all analyses were conducted on an intention-to-treat basis.

## Results

### Patient Characteristics

A total of 292 patients receiving a total of 300 consecutive procedures were included with no missing data. Surgical treatment consisted of breast-conserving surgery in 212 procedures (70.7%) and mastectomy in 86 procedures (28.7%). Two procedures (0.6%) involved nodal-only or skin procedures.

The mean age was 66.0 years (standard deviation [*SD*] 12.28), mean body mass index (BMI) was 29.09 kg/m^2^ (SD 7.09), and mean tumour size was 24.6 mm (SD 18.29), with a median tumour size of 19 mm (range 4–110 mm) and BMI ranging from 17 to 72 kg/m^2^ (median 27.95). The cohort included 115 patients (38.3%) detected through screening and 185 patients (61.7%) presenting symptomatically. Tumour grade distribution was 14.3% (*n* = 43) grade 1, 59.7% (*n* = 179) grade 2, and 26% (*n* = 78) grade 3. Based on invasive tumour size, 153 tumours (51%) were classified as T1 (<20 mm), 120 (40%) as T2 (20–50 mm), and 27 (9%) as T3 (>50 mm), demonstrating a broad range of tumour burden within the cohort. Notably, tumour size ranged from 4 to 110 mm, and BMI ranged from 17 to 72 kg/m^2^; yet neither variable significantly influenced nodal detection performance on multivariable analysis.

The nodal procedural detection rate was 99.7% (299/300), with one case requiring conversion to alternative axillary sampling. This conversion was successful and 100% of procedures retrieved pathologically identified lymph nodes.

Regarding the ICG performance, only seven procedures (2.3%) demonstrated no fluorescent nodal detection meaning that the ICG nodal detection rate was 97.7%. The mean surgeon–pathologist nodal concordance rate was 75.7% (SD 27.5), with a median concordance of 100% (IQR 50–100%). Complete agreement between surgeon-reported and pathology-confirmed node counts was observed in 152/300 procedures (50.7%). Pathology identified more nodes than were recorded intraoperatively in 124/300 procedures (41.3%), whereas surgeons reported more nodes than pathology in 15/300 procedures (5%). This likely reflects pathological subdivision of nodal tissue specimens rather than failure of intraoperative node localisation.

The mean number of nodes retrieved was 2.28 (*SD* 1.24) on pathological assessment. Nodal positivity was observed in 19.3% of patients (*n* = 58), where all pathologically identified nodes are considered or 17.3% when restricting to only SLNB related lymph nodes. Mean operative time from incision to first node identification was 7.58 min (*SD* 4.45), with a median of 6.5 min (range 1–23 min). Fluorescence was visible to the naked eye in 33% of cases, with residual fluorescent nodes identified in 1%.

Multivariable linear regression demonstrated no statistically significant association between nodal detection rate and age (*p* = 0.6587), BMI (*p* = 0.1937), tumour size (*p* = 0.2501), or surgical procedure (*p* = 0.7754), confirming independence of detection from patient and tumour characteristics. In contrast, operative time was significantly associated with BMI (β = 0.085, *p* = 0.0118) and surgical procedure (β = 3.870, *p* < 0.0001), indicating increased operative duration with higher BMI and breast-conserving surgery. Correlation analysis supported these findings, demonstrating a weak positive correlation between BMI and operative time (r = 0.11, *p* = 0.047), a strong relationship between procedure and operative time (*r* = − 0.43, *p* < 0.0001), and associations between tumour size and both procedure (*r* = 0.48, *p* < 0.0001) and nodal positivity (*r* = 0.19, *p* = 0.001), with no meaningful correlations observed between nodal detection and any patient or tumour variable (Tables 1, 2, 3 and 4).

**Table 1:** Patient characteristics

Variable	Value
Age, mean (*SD*)	66 (12.28)
BMI, mean (*SD*)	29.09 (7.09)
Tumour size (mm), mean (*SD*)	24.6 (18.29)
Tumour size, median (range)	19 (4–110)
BMI, median (range)	27.95 (17–72)
Screening presentation	115 (38.3%)
Symptomatic presentation	185 (61.7%)
Grade 1	43 (14.3%)
Grade 2	179 (59.7%)
Grade 3	78 (26%)
Breast conservation	212 (70.7%)
Mastectomy	86 (28.7%)
Nodal only	1 (0.3%)
Skin procedure	1 (0.3%)
Tumour Stage Category	
T1 (< 20 mm)	153 (51%)
T2 (20–50 mm)	120 (40%)
T3 (> 50 mm)	27 (9%)

**Table 2:** Procedural and nodal outcomes

Outcome	Value
Sentinel nodal procedural detection	299/300 (99.7%)
ICG fluorescent nodal detection	293/300 (97.7%)
Mean surgeon-pathologist concordance (%)	75.72 (27.52)
Median surgeon-pathologist concordance (%)	100
IQR	50–100
Nodes (surgeon), mean (*SD*)	1.99 (0.87)
Nodes (pathology), mean (*SD*)	2.28 (1.24)
Total nodal positivity	58 (19.3%)
SLNB restricted nodal positivity	52 (17.3%)
Operative time (min), mean (*SD*)	7.58 (4.45)
Operative time median(range)	6.5 (1–23)
Visible fluorescence	33%
Residual fluorescence	1%

**Table 3:** Regression analysis—nodal detection

Variable	Estimate	*p* value
Age	0.059	0.6587
BMI	− 0.302	0.1937
Tumour size	− 0.118	0.2501
Procedure	− 1.276	0.7754

**Table 4:** Regression analysis—operative time

Variable	Estimate	*p* value
BMI	0.085	0.0118
Procedure (BCS)	3.87	< 0.0001
Age	0.038	0.0513
Tumour size	0.002	0.8809

### Safety

No ICG-related adverse events or allergic reactions occurred.

## Discussion

This prospective study represents one of the largest evaluations of indocyanine green as a sole tracer for sentinel lymph node biopsy in breast cancer within a UK clinical setting and demonstrates near-universal procedural success and, more importantly, consistent nodal detection across a broad spectrum of patient and tumour characteristics. It addresses a key limitation of existing literature and a common concern about the reliability of single tracer techniques. The most crucial outcome of the study is the 99.7% nodal procedural detection rate, with successful identification of sentinel lymph nodes in 299 of 300 consecutive procedures operated on by five different surgeons. The ICG nodal detection rate was 97.7%, with seven procedures associated with no fluorescence. This cohort is larger than most previously reported series and is comparable to, if not superior to, traditional dual tracer approaches. Although caution is warranted when extrapolating results from a single-centre study, the study provides strong evidence to suggest that indocyanine green can achieve excellent procedural detection performance.^12,13^

A further strength of the present study is that procedures were performed by five different breast surgeons with varying levels of prior experience in fluorescence-guided sentinel lymph node biopsy. While some surgeons had extensive previous experience with ICG mapping, others were newly appointed consultants with limited exposure to the technique. Despite this variation, consistently high detection rates were achieved throughout the study period. This suggests that the learning curve associated with ICG-guided sentinel node biopsy is relatively short and supports the practical implementation of the technique across a broad range of surgical experience levels. Although procedures were performed by multiple surgeons, this was not a formal learning-curve study and surgeon-specific outcomes were not analysed.

However, an additional insight is provided by the surgeon-pathologist concordance analysis. Complete agreement between intraoperative and pathological node counts was observed in over half of procedures, while pathology identified additional nodes more frequently than surgeons. This likely reflects pathological subdivision of excised nodal tissue rather than failure of sentinel node localisation, further supporting the robustness of the technique.

A key strength of this study is the demonstration that nodal detection is independent of patient level and tumour level variables, with regression analysis showing no significant association between nodal detection rate and age, body mass index, tumour size, or surgical procedure, and correlation analysis confirming the absence of meaningful relationships between detection and these variables. This finding directly addresses longstanding concerns regarding fluorescence guided sentinel node biopsy, particularly in patients with increased body mass index or altered lymphatic drainage patterns and suggests that indocyanine green fluorescence mapping is inherently resilient to anatomical and biological variation.

This represents a significant advancement over existing evidence, which has largely been derived from smaller or retrospective cohorts,^14–16^ and provides prospective data supporting the conclusion that performance is consistent across heterogeneous patient populations, strengthening the argument for broader clinical adoption.

A central contribution of this study is the clear distinction between detection reliability and operative efficiency, as while nodal detection was unaffected by any measured variable, operative time was significantly influenced by body mass index and surgical approach, with increasing body mass index associated with longer incision to node times and breast-conserving surgery resulting in an approximate four minute increase in operative duration, indicating that the technical challenge of the procedure lies not in tracer performance but in surgical exposure and anatomical complexity. This distinction is clinically important as it demonstrates that indocyanine green remains effective even in technically demanding cases and that variations in operative time should not be interpreted as a limitation of the tracer itself but rather reflect expected differences in surgical conditions, a conclusion further supported by correlation analysis demonstrating strong relationships between procedure type and operative time and between tumour size and procedure selection, indicating that case complexity drives operative duration rather than the efficacy of lymphatic mapping.

The findings are consistent with and extend the existing body of literature, as early feasibility studies established the technical validity of fluorescence guided sentinel node biopsy and subsequent systematic reviews and meta analyses have confirmed comparable detection rates to radioisotope based techniques, although most prior studies have been limited by small sample sizes, retrospective design, or hybrid tracer use, and few have systematically evaluated the influence of patient level variables, whereas the present study provides prospective evidence demonstrating both high detection rates and independence from patient and tumour characteristics, supporting the role of indocyanine green as a standalone tracer rather than an adjunct.^17^

The current study builds upon a progressive body of evidence supporting fluorescence-guided sentinel lymph node biopsy. Early work by Kitai et al. first demonstrated the feasibility of ICG lymphatic mapping,^6^ while subsequent studies by Sugie et al.^7^ Hirche et al.^8^ and Verbeek et al.^18^ established its clinical applicability and detection performance. More recently, meta-analyses by He et al.^12^ and Zhang et al.^13^ have suggested equivalence to conventional radioisotope-based techniques. However, most published series remain retrospective, involve mixed tracer protocols, or include relatively small patient numbers. The present study extends this literature by providing one of the largest prospective evaluations of ICG as a sole tracer and, to our knowledge, represents the largest prospective UK cohort evaluating ICG-only sentinel lymph node biopsy. These findings complement those of the INFLUENCE trial and subsequent UK outcome studies while providing practical implementation data from routine clinical practice.

These findings have important clinical implications, as the demonstration of consistent detection across diverse patient populations supports the use of indocyanine green as a reliable alternative to dual tracer sentinel lymph node biopsy, particularly in settings where access to nuclear medicine services is limited, while elimination of radioisotope use simplifies logistics, reduces cost, and removes regulatory barriers, and the ability to visualise fluorescence directly enhances intraoperative usability and may reduce dependence on specialised equipment.^19^

Additionally, the distinction between detection and efficiency provides practical guidance for surgical planning, indicating that surgeons can expect consistent detection performance across patient groups while recognising that operative time may vary according to anatomical and procedural factors.

Several limitations should be acknowledged, including the single centre design which may reflect local expertise and standardised technique, the lack of long-term oncological outcomes, which remain an important area for future research, although the primary aim of the study was to evaluate performance in a real-world cohort rather than to conduct a comparative trial. Detailed classification of nodal metastatic burden into macrometastases, micrometastases and isolated tumour cells was not prospectively collected and therefore could not be analysed. Although overall nodal positivity and the number of positive nodes per patient were recorded, future studies should incorporate more granular pathological reporting to further characterise axillary disease burden. The previously published INFLUENCE^20^ trial addressed several of these methodological limitations through prospective randomised evaluation of ICG-guided sentinel node biopsy. The present study should therefore be regarded as a real-world implementation study of those findings.

## Conclusions

Indocyanine green only sentinel lymph node biopsy demonstrates robust, reliable, and consistent detection independent of patient and tumour characteristics, avoiding the logistical complexity associated with radioisotope-based mapping while eliminating the risk of blue dye-related allergic reactions and skin staining. While operative efficiency is influenced by body mass index and surgical approach, detection performance remains unaffected, supporting its use as a practical and effective single tracer strategy for sentinel lymph node biopsy in breast cancer and providing strong evidence for its adoption in routine clinical practice.
